# The Repertoires of Peptides Presented by MHC-II in the Thymus and in Peripheral Tissue: A Clue for Autoimmunity?

**DOI:** 10.3389/fimmu.2013.00442

**Published:** 2013-12-17

**Authors:** Javier A. Collado, Carolina Guitart, M. Teresa Ciudad, Iñaki Alvarez, Dolores Jaraquemada

**Affiliations:** ^1^Department of Cell Biology, Physiology and Immunology, Institut de Biotecnologia i de Biomedicina, Universitat Autònoma de Barcelona, Barcelona, Spain

**Keywords:** peptide repertoire, MHC-II, human, thymus, peripheral tissues

## Abstract

T-cell tolerance to self-antigens is established in the thymus through the recognition by developing thymocytes of self-peptide-MHC complexes and induced and maintained in the periphery. Efficient negative selection of auto-reactive T cells in the thymus is dependent on the *in situ* expression of both ubiquitous and tissue-restricted self-antigens and on the presentation of derived peptides. Weak or inadequate intrathymic expression of self-antigens increases the risk to generate an autoimmune-prone T-cell repertoire. Indeed, even small changes of self-antigen expression in the thymus affect negative selection and increase the predisposition to autoimmunity. Together with other mechanisms, tolerance is maintained in the peripheral lymphoid organs via the recognition by mature T cells of a similar set of self-peptides in homeostatic conditions. However, non-lymphoid peripheral tissue, where organ-specific autoimmunity takes place, often have differential functional processes that may lead to the generation of epitopes that are absent or non-presented in the thymus. These putative differences between peptides presented by MHC molecules in the thymus and in peripheral tissues might be a major key to the initiation and maintenance of autoimmune conditions.

## Peptide Repertoire in Thymus

Differences between the peptide repertoires associated to MHC-II molecules expressed by antigen presenting cells (APC) in the thymus and in the periphery have been long proposed, but are yet to be demonstrated (1). Such differences were expected after the characterization of the different thymus APCs, some of which have unique properties of their antigen processing machinery, compared to peripheral APCs (2, 3). The earliest analysis of the MHC-associated peptide repertoires in the thymus and spleen was done in the mouse in the 90s (4), showing similar repertoires, but the technological limitations of the time failed to provide a sufficient number of peptides. The current technology and computer tools have allowed better yields, providing a broader view of the *in situ* presented peptides and in-depth study of the characteristics and general composition of the MHC-associated peptide repertories.

We have recently described the peptides presented by HLA-DR molecules derived from pediatric thymus samples in homeostatic conditions, that included the peptides presented by all thymus APCs (5). As expected, the total expression of HLA-DR was at least three times higher in the medulla than in the cortex region (6). In this context, a major fraction of the described repertoire would be medulla-derived and might thus represent the most abundant peptides presented in thymus during negative selection. Whether medullar or cortical, our data showed a thymus repertoire composed of a diverse number of peptides derived from a wide range of proteins, i.e., not a highly redundant repertoire. Proteins involved in most frequent functional events occurring in the thymus, i.e., apoptosis and antigen processing, were major providers of peptides for their presentation by HLA-DR. The apoptosis-related peptides may come from the uptake of apoptotic cells (mostly thymocytes) by APCs (7, 8) or directly from the AutoImmune REgulator (AIRE)-induced mTEC apoptosis (9–11). The remaining source proteins were of high diverse origin, including nuclear and cytosolic proteins, conventionally considered as belonging to the Class I – endogenous pathway. Most were conventional size MHC-II peptides from proteins derived from membrane-rich compartments. Many were grouped into nested sets and most represented a single region of the parental protein at the expense of the rest of the sequence. Thus, these data reveal that tolerance against a protein is usually developed against a specific region, rather than to many epitopes of the protein.

In a nearly simultaneous paper, Adamopoulou et al. (12) reported the analysis of the thymus MHC-I and -II-associated peptidome. This work analyzed DC and DC-depleted APCs from the thymus separately, whereas our study included the peptides presented by all thymus APCs, preferentially medullary APCs. The results by Adamopoulou et al. essentially agree with our data, confirming a large number of source proteins of diverse origin and a clear “cross-talk” between the MHC-I and -MHC-II pathways of antigen processing. Recent results from our group on the MHC-I-associated thymus peptidoma (13) show similar results including the presentation of 20% peptides derived from proteins processed by the exogenous pathway. Thus, the “non-conventional” pathways of MHC-I and -II processing, i.e., cross-presentation of exogenous antigens by MHC-I and autophagy and uptake of apoptosis material for the presentation of cytosolic proteins by MHC-II, are efficiently acting in the thymus. It remains to be analyzed which of the thymus APCs are more involved in these pathways.

In addition to HLA-DR, HLA-DM expression is also weaker in the thymus cortex than in the medulla (6). HLA-DM is needed for the high-stability binding of peptides to HLA-DR. HLA-DM forms stable complexes with DR molecules following CLIP dissociation at low pH and allows the formation of stable peptide-MHC (pMHC) complexes (14, 15). Thus, peptides presented in the medulla should be high-stability peptides forming stable complexes (16). We observed that most HLA-DR ligands identified *ex vivo* from the thymus showed high predicted affinity for one or both of the expressed HLA-DR alleles (5). In addition, the 12 allele-assigned peptides in the Adamopolou report (6 DRB1*03, 6 DRB1*04) were also high binders when analyzed by the panMHC-II prediction software (17) (J. A. Collado, data not shown).

The thymus can generate a large variety of MHC ligands for display through activities like highly activated thymocyte proliferation and apoptosis, constitutive levels of autophagy by TECs (18, 19), antigen exchange between mTEC and DCs (20) or expression of tissue-restricted antigens (TRA), by mTEC (21). The resulting peptides will necessarily compete for *de novo* synthesized MHC-II molecules. This constant competition should presumably favor the presentation of the highest affinity ligands, displacing those peptides with lower affinities for the binding groove. High-stability peptides form long-life pMHC complexes at the APC surface, increasing the possibility of recognition by self-reacting CD4^+^ thymocytes and improving negative selection efficiency. In contrast, low-stability complexes are short-lived, so they may be ignored or only be recognized by a small number of thymocytes, increasing the probability of escaping selection.

## MHC-II Peptide Repertoire in Autoimmunity

Secondary lymphoid organs are essential for the maintenance of tolerance so MHC molecules should present much the same peptides as those presented in thymus. Our analysis of a spleen HLA-DR repertoire showed many common peptides with an HLA-DR-matched thymus, and with slight differences related to the spleen function such as a large number of peptides from blood proteins (Collado et al., unpublished). However, few studies are available concerning the peptide repertoires associated to MHC-II in healthy lymphoid tissue (4, 22).

In contrast, there are more data describing the MHC-II peptidome from non-lymphoid peripheral tissues, affected by autoimmunity (23–25) or cancer (26). These were composed of peptides that reflected the characteristic protein composition of each organ. Albeit most peptides were from ubiquitous proteins, there were sequences derived from tissue-restricted proteins potentially related to the disease. We were the first to demonstrate the *in vivo* presentation of thyroglobulin (Tg) peptides by HLA-DR in thyroid glands affected by Graves’ disease (23). In later reports, peptides from myelin basic protein (MBP) and other autoantigens were identified in central nervous system (CNS) samples from multiple sclerosis (MS) patients (24), and peptides from collagen, vimentin, and others were detected in synovial samples from rheumatoid arthritis (RA) patients (25). Thus, tissue-expressed MHC-II molecules displayed autologous peptides, including some from known autoantigens. These proteins are abundant in these tissues, and there should be enough specific-pMHC complexes *in situ* to stimulate potential auto-reactive T cells. In our study, we identified eight peptides from different regions of Tg, a major component of the thyroid colloid and a common autoantigen in autoimmune thyroid diseases (27). Similarly, up to six different MHC-II-associated MBP peptides were identified from human MS patients’ CNS (24) and several peptides from collagen in RA patients (25). In contrast, the peptides identified in the thymus in homeostatic conditions mostly came from one single region of each protein. This suggests that a wide range of peptides derived from the same protein may be generated and presented in the autoimmune target tissue, whereas efficient tolerization in the thymus may be only directed against the dominant epitope or epitopes of each protein.

Furthermore, when we analyzed the potential affinity of peptides from autoimmune thyroids, only one third of the sequences corresponded to peptides with predicted high affinity for HLA-DR (5). Only one out of the eight Tg sequences identified was assigned to any HLA-DR allele, i.e., Tg2098, an immunodominant Tg peptide (23, 28, 29). The remaining peptides were low-affinity HLA-DR ligands, both according to predicted and experimental binding data (5, 23). A prediction analysis of the affinity of CNS peptides from MS patients (24) again showed less than 20% of the peptides with high affinity and around 65% low-affinity peptides (J. A. Collado, data not shown). So, contrary to thymus, where low-affinity peptides appeared to be relatively unavailable for presentation by HLA-DR, these peptides were abundant in the affected tissue.

Thus, there are differences between the repertoires in thymus and autoimmune-affected tissue: (i) no apparent immunodominance of any particular region of each protein, contrary to that observed in thymus and (ii) low affinity of many autoantigenic peptides generated in the peripheral tissue for the MHC-II molecules. Some of these peptides, able to bind MHC with lower affinity, would be good candidate targets of the autoimmune response.

## Mechanisms Generating Repertoire Differences

Specific cellular processes in peripheral tissues may modify the outcome of antigen processing and presentation *in situ*. This can favor the generation of peptides that would have been ignored or not generated in the thymus. Several mechanisms may be involved:

Tissue-specific proteolytic machinery affecting proteins before uptake by APCs.
High concentration of tissue-specific antigens in the target tissues.
Post-translational modifications of proteins in the tissues.
Expression of HLA-DR and DM in autoimmunity affected tissue.
T cells exclusive for tissue-derived peptides.

### Proteolysis

Normal turn-over of tissue-specific proteins, cell death, extracellular processing, together and different tissue proteases may provide a source of peptides that will not be found in thymus. In the thymus, lysosomal proteases and enzymes involved in antigen presentation are diverse cathepsins (Cat), asparagine endopeptidase (AEP), and Gamma-interferon-inducible lysosomal thiol reductase (GILT). Detailed cathepsin expression by each particular cell subset of the human thymus has been recently reported (2). Cat V is expressed only in the cortex while Cat L is expressed by few cells distributed throughout the thymus (30). cTEC and mTEC express all the studied cathepsins (B, D, H, S, and X) and GILT. Mature DCs expressed the highest levels of those cathepsins (especially Cat S). However thymus DCs did no express Cat G in contrast to peripheral blood DCs (31). The importance of cathepsins in tolerance is evidenced when autoantigen processing is studied. Cat S processes MBP in thymus DCs generating an immunodominant epitope that is destroyed by Cat G in peripheral blood DCs. Cat S cleavage of proinsulin can also destroy insulin T-cell epitopes (2). Moreover, NOD mice deficient in Cat B, S, or L are protected from type I diabetes development (32).

Besides the different cathepsins expressed by tissue APCs, extracellular matrix remodeling or partial degradation of stored proteins in the tissue milieu are another source of neo-epitopes. The cleavage of Tg in the thyroid is a good example. Tg is produced by thyroid follicular cells (TFC) and secreted into the colloid. Solubilization and pre-cleavage of Tg are necessary prior to endocytosis by TFC to generate T3 and T4 hormones. Cat B, L, S, K present both in colloid and in the TFC’s endocytic vesicles cleave Tg at different pH (neutral and acid pH, respectively) (33), giving a different pattern of cleavage in each condition. Moreover, overexpression of cathepsin B in the thyroid has also been reported in autoimmune thyroid diseases (34). Similar extracellular matrix remodeling occurs in other autoimmune diseases, such as RA, where Cat S, K, B, and L are secreted to synovial fluid and tissue (35, 36). Thus, the differential processing by APCs may be relevant when comparing thymus and autoimmune peripheral tissue. A wide range of different peptides can be generated in the affected tissue, leading to the activation of auto-reactive T cells that were not negatively selected, causing and maintaining the autoimmune process.

### Differential dose of antigen in peripheral tissue vs. thymus

The expression of TRAs in the thymus is temporally regulated and is weaker than in the corresponding peripheral tissue (37). Major components of specific organs, such as insulin in the pancreatic islets or Tg in the thyroid gland, are expressed at low levels in the thymus by a very small subset of HLA-DR^+^AIRE^+^ mTECs (38, 39). This suggests that to generate tolerance, a small number of TRA peptides should be sufficient. However, quantitative and qualitative variability of the presented peptides may influence the number of auto-reactive T cells that exit the thymus. In humans, the T1D-associated *IDDM2* susceptibility locus of the insulin gene (*INS*) contains variable number of tandem repeat (VNTR) polymorphisms upstream to the *INS* promoter. The length of these repeats is directly involved in the control of the expression levels of insulin mRNA in the thymus (40). A polymorphism of the thyroid stimulating hormone receptor (TSHR) gene, with susceptible and protective alleles corresponding to low and high expression of the gene in the thymus and the target organ has also been described (41). In a mouse model of thyroid autoimmunity, transgenic BALB/c mice were generated with the human TSHR A-subunit targeted to the thyroid and thymus. Two types of mice were obtained, low and high TSHR expressors. When these mice were immunized with a human TSHR construct and Treg cells were depleted, the low-expressors suffered the disease (thyroid infiltration and damage) while the high expressors remained tolerant. Presumably, THSR peptides presented in the high expressors’ thymus were enough to induce tolerance to all possible epitopes, whereas low-expressors presented insufficient TSHR peptides or presented them with low efficiency. These peptides would be presented in periphery and responsible for the *in situ* reactivity (42).

Therefore, peptides from all relevant antigens in the body should be present in the thymus at an adequate concentration to prevent autoimmunity. No data are as yet available of DR-associated peptides from TRAs presented in the thymus, although peptides from non-organ-specific proteins that are potential targets of autoimmune disease have been described (12) and we have some data suggesting the presence of TRA-specific peptides in thymus samples (Alvarez and Collado, unpublished data).

### Post-translational modifications in the tissue

Other local events likely involved in the development of autoimmunity are post-translational modifications (PTMs) common in most mammalian proteins that allow the generation of neo-self epitopes (43, 44). PTMs can occur spontaneously or arise by enzymatic modification, altering the protein structure, and biological functions. Modifications of the proteolytic degradation can also occur. PTMs that generate neo-self-peptides include enzyme-dependent glycosylation (45), deamidation (46), citrullination (47, 48), iodination (49), phosphorylation (50), methylation (51), or chemical modifications such as disulfide bridge formation, oxidative modification or nitration, and many others (52).

Many of these PTMs have been associated with one or more autoimmune processes (53). One of the best studied PTM is citrullination, the substitution of arginine by citrulline. Citrullination increases the sensitivity of MBP to cleavage by cathepsin D, allowing epitope destruction or neo-epitope generation (54–56). In RA, citrullination may play a role in T-cell autoreactivity. RA-associated HLA-DR molecules share a consensus sequence in the peptide binding groove, named the “shared epitope” (SE) (57), that contains a positively charged P4 peptide binding pocket. Citrullination will remove a positively charged Arg residue from any peptide, enhancing its ability to bind to SE-MHC-II molecules. In a mouse model, the substitution of Arg by Cit in a candidate vimentin peptide efficiently immunized DRB1*0401 transgenic mice, whereas the original peptide did not. This analog showed markedly enhanced binding to SE-HLA alleles, but not to other alleles (52).

Thyroglobulin is extensively modified by iodination and other post-translational events in the thyroid colloid. Iodinated Tg epitopes are highly immunogenic and can trigger thyroid auto-reactive T cells (49, 58). Other examples include the inhibition of the generation of the 85–99 dominant epitope of MBP by processing enzyme AEP in the thymus, thus limiting the recognition of this epitope by thymocytes. AEP is not highly expressed in CNS (59).

### Expression of HLA-DR and DM in autoimmunity affected tissue

In organs affected by autoimmune diseases, cell targets of the tissue damage often overexpress MHC-I and ectopically express MHC-II molecules (59–64). This MHC expression is clearly related to the maintenance or even the induction of self-reactive responses, although the mechanisms involved are not known.

The importance of this expression in the pathology may vary in the different tissues. There is very high expression of MHC-II in autoimmune TFC, whereas class II expression in pancreatic islets in diabetes is not so clear, despite the stronger association to HLA-DR and -DQ alleles with T1D compared to thyroid autoimmunity (65). HLA-DM is also expressed by autoimmune TFC, although at lower levels than in conventional APCs (66). In the absence of DM or if DM is insufficient, high affinity peptides may be outcompeted by low-affinity peptides.

In addition, characteristics of particular alleles associated with disease may also influence the autoimmune processes. In the mouse, it is well known that H-2 I-A^g7^ of the NOD mouse is a weak peptide binder with a highly specific binding motif (67). In human, the HLA-DR3 allele, associated with a wide range of autoimmune diseases, has low affinity for CLIP (68). Thus, DR3 may not so strictly require DM to ensure peptide presentation, avoiding efficient peptide selection and allowing the binding of low-stability peptides (69). HLA-DQ2, of the “autoimmune” HLA-DR3-DQ2 haplotype, associated with susceptibility to T1D and celiac disease, also interacts poorly with DM (69, 70). DQ2/DQ8 heterozygous individuals, highly susceptible to T1D, can form transdimers HLA-DQ2α/DQ8β, capable of presenting different peptide repertoires than their *cis* counterparts (71). It is not known whether transdimer formation in the thymus is as efficient if at all, as it is in the affected tissues.

### Unique T-cell recognition of tissue-derived antigens

It has been reported that peptides from antigens expressed in tissues can be presented by local APC activating self-reactive T cells. Evidence comes from studies that show that APCs stimulate a subset of T cells only when they are loaded with soluble peptide but not with the whole protein (72). These so-called type B T cells recognize pMHC complexes that are different from those generated when peptide is derived from intracellular processing of native proteins. One reasonable explanation is that peptides can bind MHC-II molecules on plasma membrane and also in recycling vesicles, avoiding the effects of HLA-DM, so less-stable complexes are available for presentation. Moreover, register shifting of peptides at the binding groove might be relevant since loading of exogenous peptide could lead to different conformations. Such T cells have been reported to infiltrate islets of pre-diabetic NOD mice (73, 74) and to escape from tolerance in EAE (75).

## Concluding Remarks

**Figure 1:**
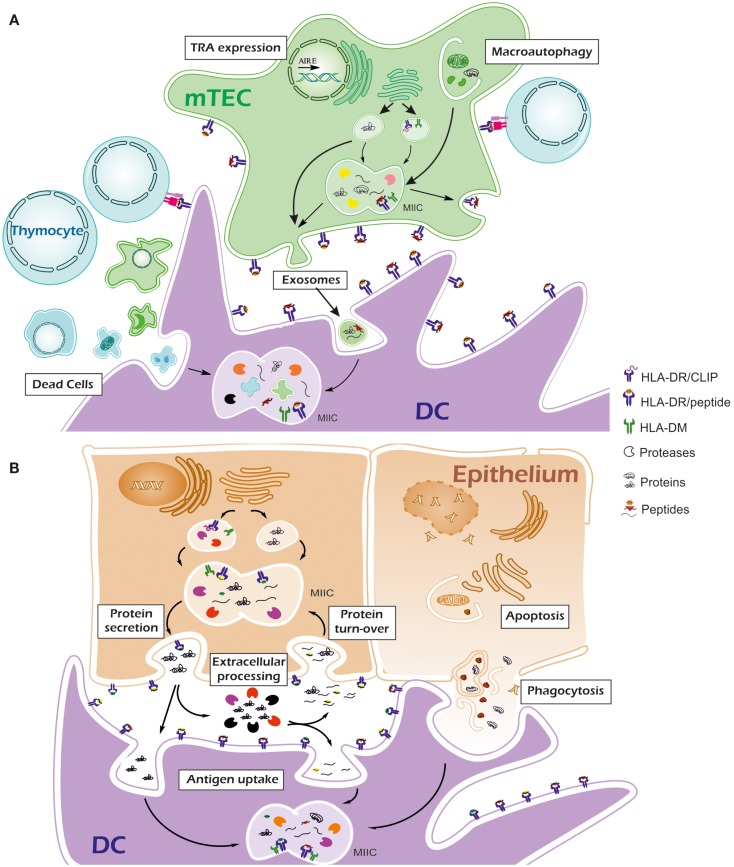
**Thymus and peripheral tissue mechanisms involved in antigen processing**. **(A)** In thymus, tissue-restricted antigens (TRAs) are expressed by medullary epithelial cells (mTECs) for negative selection. Secretory TRAs are processed by self-proteases and peptides (orange squares and red triangles) are loaded onto MHC-II molecules once exocytic vesicles reach the MIIC compartment. Peptide exchange is mediated by HLA-DM. Macroautophagy permits the access of cytosolic TRAs and other proteins into the MIIC compartment so their processing and presentation via MHC-II can take place. Besides their own proteome, thymus dendritic cells (DC) are expected to present antigens via the uptake of apoptotic thymocytes and mTECs, exosomes delivered by mTECs and other extracellular material. Thymus-specific and conventional proteases generate high affinity peptides from a wide range of self-proteins, thus favoring their presentation in competition with low-affinity peptides. **(B)** In peripheral tissues, specific proteases may be essential for peptide generation. A protein can generate peptides for MHC-II in the secretory pathway. Tissue-specific post-translational modifications may result in modified antigenic peptides (orange squares from thymus are represented as green squares in periphery) or even prevent the generation of some peptides. Once in the extracellular environment, proteases could partially degrade the protein into smaller fragments (yellow circles) that would be potential binders for MHC-II molecules. In some cases protein storage outside the cells (e.g., thyroglobulin in thyroid colloid) is followed by protein turn-over into the cells. Proteins or their cleavage products contained in endosomes can be processed again in the MIIC compartment to generate MHC-II ligands. During inflammation, infiltrating DCs can uptake the antigenic proteins or their fragments for processing with their specific proteases. They can also phagocyte apoptotic epithelial cells. Compared with thymus, events in periphery may result in a set of presented peptides that were not used in negative selection. Lack of competition with high affinity peptides and low or absent expression of HLA-DM may result in the presentation of peptides with low affinity for MHC-II molecules.

One of the clues to autoimmune processes may lay in the differences of the peptide repertoire associated to MHC-II molecules between thymus and the target tissue. Tissue-specific conditions, including proteolysis, PTMs of proteins and high expression of target antigens, together with the specific properties of the disease-associated alleles, may generate an *in situ* repertoire with enough differences from the repertoire in thymus to activate a good number of otherwise low-affinity auto-reactive T cells. See Figure 1.

## Conflict of Interest Statement

The authors declare that the research was conducted in the absence of any commercial or financial relationships that could be construed as a potential conflict of interest.
